# *Clostridium difficile* infection: review

**DOI:** 10.1007/s10096-019-03539-6

**Published:** 2019-04-03

**Authors:** Jacek Czepiel, Mirosław Dróżdż, Hanna Pituch, Ed J. Kuijper, William Perucki, Aleksandra Mielimonka, Sarah Goldman, Dorota Wultańska, Aleksander Garlicki, Grażyna Biesiada

**Affiliations:** 1 Department of Infectious and Tropical Diseases, Jagiellonian University, Medical College, Śniadeckich 5, 31-501 Krakow, Poland; 2 Saint Ann’s Hospital, Miechów, Poland; 3 Department of Medical Microbiology, Medical University of Warsaw, Warsaw, Poland; 4 Department of Medical Microbiology, Centre for Infectious Diseases, Leiden University Medical Center, Leiden, The Netherlands; 5 Centre for Infectious Diseases Research, Diagnostics and Laboratory Surveillance, RIVM, Bilthoven, The Netherlands; 6 Department of Medicine, John Dempsey Hospital, University of Connecticut, Farmington, CT USA; 7 University Hospital, Krakow, Poland

**Keywords:** Antibiotic-associated diarrhea, *Clostridium difficile*, Diagnosis, Fecal transplantation, Treatment

## Abstract

*Clostridium difficile* (*C. difficile*) is a Gram-positive, spore-forming, anaerobic bacillus, which is widely distributed in the intestinal tract of humans and animals and in the environment. In the last decade, the frequency and severity of *C. difficile* infection has been increasing worldwide to become one of the most common hospital-acquired infections. Transmission of this pathogen occurs by the fecal-oral route and the most important risk factors include antibiotic therapy, old age, and hospital or nursing home stay. The clinical picture is diverse and ranges from asymptomatic carrier status, through various degrees of diarrhea, to the most severe, life threatening colitis resulting with death. Diagnosis is based on direct detection of *C. difficile* toxins in feces, most commonly with the use of EIA assay, but no single test is suitable as a stand-alone test confirming CDI. Antibiotics of choice are vancomycin, fidaxomicin, and metronidazole, though metronidazole is considered as inferior. The goal of this review is to update physicians on current scientific knowledge of *C. difficile* infection, focusing also on fecal microbiota transplantation which is a promising therapy.

## Introduction

### CDI has become one of the most significant nosocomial infection

*Clostridium difficile* (*C. difficile*) is a Gram-positive, anaerobic, spore-forming, toxin-producing bacillus, which was officially renamed in 2016 to *Clostridioides difficile.* New name reflects the taxonomic differences between this species and other members of the *Clostridium* genus [1, 2]. Spores of *C. difficile* are transmitted by the fecal-oral route, and the pathogen is widely present in the environment. Potential reservoirs for *C. difficile* include asymptomatic carriers, infected patients, the contaminated environment and animal intestinal tract (canine, feline, porcine, avian). Approximately 5% of adults and 15–70% of infants are colonized by *C. difficile*, and the colonization prevalence is several times higher in hospitalized patients or nursing home residents [3]. *C. difficile* was first isolated from a healthy newborn’s stool in 1935 by Hall and O’Toole [4]. Until the 1970s, it was considered as a microorganism that is rarely, but present in normal intestinal microbiota. After the introduction of antibiotics, the role of *C. difficile* in the pathogenesis of large intestine diseases increased. In 1974, Tedesco et al. found that 21% of patients treated with clindamycin developed diarrhea. Pseudomembranes were found in 50% of cases, as revealed by further endoscopic examination [5]. At the end of the twentieth century, the incidence of *Clostridium difficile* infection (CDI) markedly increased. Currently, CDI has become one of the most significant nosocomial infections, which affects all hospital wards.

## Risk factors associated with CDI

### Antibiotic exposure, older age, and hospitalization are key factors for CDI development

Significant patient-related risk factors for CDI are antibiotic exposure, older age, and hospitalization. Nearly every antibiotic has been associated with the development of CDI, including the drugs used for treatment of CDI: metronidazole and vancomycin. Broad spectrum penicillins and cephalosporins, clindamycin, and fluoroquinolones possess a higher risk for CDI induction than other antibiotics [3]. The risk for development of CDI is 8- to 10-fold higher during antimicrobial therapy and 4 weeks thereafter, and 3-fold higher in the next 2 months [6]. Patient age > 65 years increases the risk for CDI 5 to 10-fold, compared with patients < 65 years of age. Nonetheless, a significant proportion of CDI occurs in a younger population. Age > 65 years is a significant risk factor not only for CDI itself, but also for poor clinical outcome including severity and mortality [3, 7]. Although most cases of CDI are linked to healthcare exposure, either hospitalization or nursing home stay, recent studies suggest that the incidence of community-acquired CDI is growing, and might have recently reached up to 30% of all CDI cases [8]. The percentage of hospitalized patients with *C. difficile* colonization differs by country, patient age group, and length of hospitalization. During the first days of hospitalization, the incidence of *C. difficile* colonization ranges from 2.1 to 20% [9–13], and increases with longer hospital stay, e.g., from 20 to 45.4% in a study by Huang et al., from 2.1 to 50% after 1 month of hospitalization in a study by Clabots et al., and from 1 to 50% after > 1 month of hospitalization in a study by Johnson et al. [12, 14, 15]. It must be noted that colonization does not necessarily mean symptomatic infection; it is suggested that only 25–30% of asymptomatic colonized patients develop diarrhea. *C. difficile* spores survive in the environment for several months [16]. Toilets, clinic furnishings, phones, and medical devices (thermometers, stethoscopes) may all serve as reservoirs for the *C. difficile* spores. The spores can be transferred to patients via the hands of healthcare personnel; therefore, good hand hygiene with soap and water and regular vinyl glove use is crucial to interrupt the transmission, as demonstrated by Johnson et al. [17]. Nursing home residents are at higher risk for CDI than the overall population, but lower than hospitalized patients (15%). This is mainly due to older age, comorbidities, more frequent hospitalizations, and more frequent antibiotic therapy in this group compared to the non-institutionalized population. *C. difficile* is the most common cause of nosocomial diarrhea [18]. It has been postulated that gastric acid suppression may have an influence on CDI development, but subsequent analysis adjusted for other comorbidities did not confirm this hypothesis [19, 20]. This is in line with the observations that gastric acid did not kill the *C. difficile* spores [3]. Nonetheless, this topic remains controversial, as several studies and meta-analyses have found a significant association [21–23], whereas other have failed to associate proton pump inhibitors use with risk of CDI development [24–26].

Other well-defined risk factors for CDI include inflammatory bowel disease, gastrointestinal surgeries, immunological incompetence caused by malignant neoplasms, transplantations, chronic kidney diseases, or immunosuppressant use [3, 27].

## Pathogenesis

### The main protective barrier against CDI is the normal intestinal microflora

Infection with *C. difficile* mostly occurs as a result of spore transmission. Spores are resistant to heat, acid, and antibiotics. The main protective barrier against CDI is the normal intestinal microflora. After reaching the intestine, bile acids play an important role in the induction of *C. difficile* spore germination [28]. Bile acids are cholesterol derivatives produced and transformed in the liver. They facilitate the absorption of fats and fat soluble vitamins in the intestine. They support digestion, improve gastrointestinal motility, and actively affect bacterial flora [29, 30]. We distinguish between primary and secondary bile acids. Primary bile acids, cholic acid and chenodeoxycholic acid, are synthesized in the liver from cholesterol and are secreted into the intestine after eating. Then, as a result of active transport, about 95% of primary bile acids are reabsorbed in the final part of the intestine. The remaining, non-reabsorbed bile acids may undergo 7a-dehydroxylation by gut bacteria, transforming into secondary bile acids, deoxycholic acid and lithocholic acid [30]. In vitro, primary bile acids generally stimulate germination of *C. difficile* spores; the secondary bacteria inhibit this process [31, 32]. Moreover, in patients with CDI, there are changes in the fecal content of bile acids. Allegretti et al. showed a higher concentration of secondary bile acids in the feces of healthy people compared to CDI, while primary bile acid concentration was higher in patients with recurrent CDI compared to patients with their first episode of infection [33]. However, it has to be reinforced that the influence of bile acids is likely more complex than the simple model where primary bile acids strictly promote and secondary bile acids inhibit *C. difficile* germination and vegetation as described in the excellent review by Baktash et al. [29].

When the balance of gut microorganisms is disrupted, *C. difficile* starts to dominate and colonize the large intestine which might be the first step of infection. As mentioned previously, only a portion of colonized patients will develop symptoms of CDI [3]. The pathogen is not invasive, and virulence is mostly due to enzymes, such as collagenase, hyaluronidase, chondroitin-sulfatase, as well as toxins, which damage the epithelial cell cytoskeleton, leading to disruption of tight junctions, fluid secretion, neutrophil adhesion, and local inflammation. The result is a breakdown of gut barrier integrity and loss of functionality [29, 34]. *C. difficile* produces two important in disease pathogenesis types of toxins, A and B, which are both enterotoxic and cytotoxic; however, traditionally, toxin A is named “enterotoxin A” and toxin B, “cytotoxin B.” *C. difficile* transferase (CDT; or binary toxin) is a third toxin produced by some *C. difficile* strains, including the epidemic PCR ribotypes 027. It probably can form microtubule-based protrusions on epithelial cells, which theoretically could have a clinical impact. There are reports of severe CDI development caused by the TcdA^−^TcdB^−^CDT^+^ strain [35].

Toxins are transported to the cell cytoplasm, where they inactivate the Rho family of GTPases. The Rho protein takes part in actin polymerization, and therefore stabilizes the cell cytoskeleton. As a result of Rho protein inactivation, the inflammatory process intensifies. In more severe cases, microulcerations covered with pseudomembranes (composed of destroyed intestinal cells, neutrophils, and fibrin) start to occur on the intestinal mucosal surface. Initial studies on animal models suggested that toxin A plays a dominant role, and the action of toxin B may occur only via the tissue damage caused by toxin A [36]. However, in studies involving human colonic tissue, TcdB was a potent inflammatory toxin, whereas TcdA was even weaker, and both toxins were able to elicit CDI symptoms independently [36–38].

The *C. difficile* BI/NAP1/027 strain is hypervirulent and resistant to fluoroquinolones, exhibits intensive spore production, and is responsible for the most severe CDI cases. The *C. difficile* BI/NAP1/027 epidemic strain is characterized by two mutations in the toxin regulatory gene *tcdC*, an 18 base-pair (bp) deletion, and deletion at position 117, which leads to increased production of toxins A and B [3, 39]. It was first isolated at the beginning of the twenty-first century in North America and Europe. BI/NAP1/027 was extremely rare before 2000; in the first two large epidemics of CDI in North America at the beginning of the last decade, the percentage of CDI caused by BI/NAP1/027 was 51% in the US and 84% in Canada [39, 40]. Analyzing the data of 6000 CDI cases prior to 2001, only 14 cases attributed to BI/NAP1/027 were identified, representing only 0.2% of all cases [40]. Furthermore, numerous cytokines play a role in CDI pathogenesis, including IL-8, IL-1β, IL-6, TNFα, INFγ, and leukotriene B4 [41–43].

## Clinical manifestation

### CDI clinical picture can vary from the asymptomatic carrier state to life-threatening colitis resulting with death

The clinical picture of CDI is very heterogenous, and ranges from the asymptomatic carrier state, mild or moderate diarrhea, to life-threatening fulminant colitis. Although the incubation period is not precisely defined, and some reports suggest 2–3 days, more recent studies demonstrate that the incubation period might be even longer than 3 days and is very individual-dependent [44–47]. CDI can affect every part of the colon, but the distal segment is most commonly infiltrated. Most patients with CDI suffer from mild diarrhea and experience recovery spontaneously after 5–10 days of antibiotic therapy withdrawing. Diarrhea occurs in most cases during, or directly after antimicrobial therapy, although CDI onset might be also a couple of weeks afterwards. The clinical features of CDI, in addition to watery diarrhea, include abdominal pain, fever, nausea and vomiting, weakness, and loss of appetite. Fecal occult blood test is often positive, although active bleeding is rarely present [47]. In the most severe clinical presentation of CDI, symptoms are life-threatening, and include significant dehydration, abdominal distension, hypoalbuminemia with peripheral edema, and subsequent circulatory shock. Other severe complications of CDI include toxic megacolon, colon perforation, intestinal paralysis, kidney failure, systemic inflammatory response syndrome, septicemia, and death [47]. Extracolonic manifestations of CDI are rare, and most commonly involve small intestine infiltration, reactive arthritis, and bacteremia [43]. Mortality rate directly due to CDI is estimated at 5%, whereas mortality associated with CDI complications reaches 15–25%, and up to 34% in intensive care units (ICU). Mortality doubles in ICU patients with CDI, as compared with ICU patients without CDI [7, 48, 49]. Poor outcome is associated with older age, high leukocytosis, hypoalbuminemia, and high creatinine level [43, 50]. It has been also shown that first-ever CDI episode increases the overall risk of death [7].

CDI relapse of symptoms occur most commonly during the first week after the initial episode when treatment is complete. After effective treatment of first CDI episode, at least one new recurrent episode occurs in 10–25% of patients, and up to 65% in patients who experienced already > 1 recurrent CDI [51, 52]. There is evidence to show that half of the recurrent CDI cases are due to relapses of infection with the original strain, whereas the other half is caused by re-infection with different strains. Impaired immune response to *C. difficile* toxins, as well as new exposure to spores, is thought to contribute to recurrences. Antibiotic resistance does not seem to influence the risk of recurrences [18, 43, 53].

## Prevention

### Prevention strategies should be implemented in every suspected case, not only in confirmed patients

Strategies for prevention of CDI include the use of gloves and disposable gowns by healthcare personnel and visitors during the whole diarrheal episode. After every direct contact with a CDI-patient, everyone should wash their hands with soap and water. Alcohol-based hand hygiene products do not damage the *C. difficile* spores, whereas the mechanical hand washing with the use of running water and soap prevents spread of the spore. Optimally, every patient with CDI should be isolated in a single room. If this is not possible, contact between patients should be avoided, (e.g., reading the same books/magazines, using the same phone), and the patient should have his or her own furnishing. There are no current recommendations to screen asymptomatic carriers, as effectiveness has not been proven. Chlorine-based solutions are commonly recommended for environmental cleaning, with 1000 ppm of chlorine concentration being effective, and 5000 ppm being the most optimal choice [54]. Prevention strategies should be implemented in every suspected case, not only in confirmed patients. After discharge, the patient’s room should be carefully decontaminated [3].

## Diagnosis

### No single test is suitable as a stand-alone test confirming CDI

CDI should first be considered when diarrhea symptoms are present (≥ 3 loose stools during 24 h). The diagnosis of CDI is based on detection of *C. difficile* toxins directly in a stool sample, most commonly with an enzyme immunoassay (EIA), which provides rapid turnaround time (about 1–2 h), as well as sensitivity of 75–85% and specificity of 95–100%. Because of its low cost and ease of use, this is the most popular test in all laboratories. Tests detecting *C. difficile* antigens are based on the detection of glutamate dehydrogenase (GDH) and are characterized by ease of use and rapid turnaround time as well as a specificity of almost 100%. However, they do not distinguish whether the strain is toxigenic (specificity of 59%) [18, 55]. It should be pointed out that old-generation assays (using latex agglutination) had sensitivity of 58–68% and specificity of 89–99%. In 2009, tests that use amplification of nucleic acid (NAAT, nucleic acid amplification test) were introduced. They are based on either a PCR method or isothermal amplification. NAAT have higher sensitivity (80–100%) and specificity (87–99%) compared to an EIA test. The specificity is especially high, reaching 95%, when a negative result is obtained. In this situation, another cause of diarrhea should be considered [47, 56, 57]. The NAAT have also limitations, namely, high cost and some interpretation difficulties. PCR detects the presence of a toxin encoding gene, thus confirms the presence of *C. difficile* toxin-producing strain, but it does not necessarily mean that the strain produces any toxins at the moment. If the diarrhea is of other origin, detection of such strain would become misleading, as it would pursue further treatment towards CDI. Persistent and often ineffective treatment of only colonized patients does not improve their clinical situation. When dealing with such diagnostic difficulties, comprehensive diagnostic evaluation of other potential diarrhea causing disorders is required. A cytotoxic assay test (CYTA) is not routinely used in microbial culture due to its slow turnaround time and lack of standardization (48–72 h) [3, 47].

**Fig. 1:**
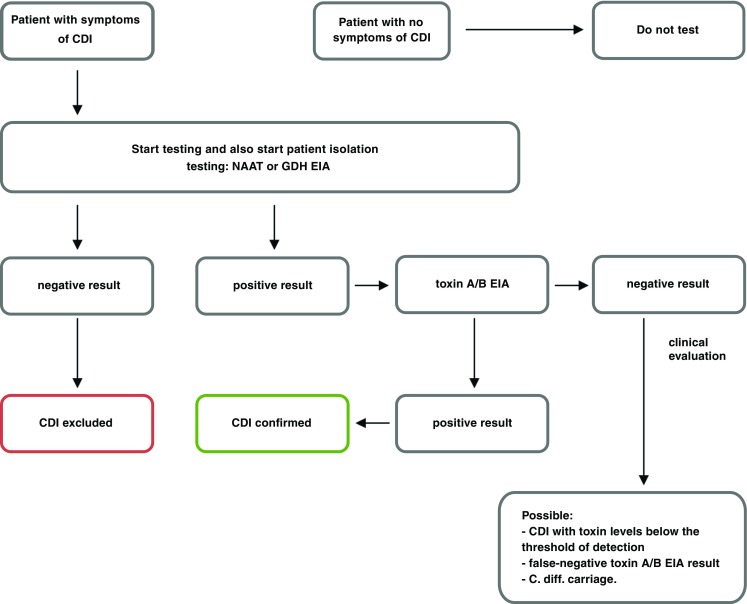
Flow chart of CDI diagnosis

According *European Society of Clinical Microbiology and Infectious Diseases* (ESCMID) guidance, no single test is suitable as a stand-alone test confirming CDI. The best way to optimize diagnosis of CDI is to combine two tests in algorithm. The first test should be a test with high negative predictive value (it can either be a GDH EIA or NAAT). The second test should be a test with a high positive predictive value (it is toxin A/B EIAs). If the first test is negative, it excludes CDI. If the first test is positive, the second test (toxin A/B EIAs) should be performed. If the second test is positive, it confirms CDI. If the second test is negative, the case needs to be clinically evaluated, and such result can be seen in three situations: CDI with toxin levels below the threshold of detection, false-negative toxin A/B EIA result, or *C. difficile* carriage. Samples with a negative GDH result but that are positive for toxin need to be retested, as this is an invalid result [58]. Flow chart of CDI diagnosis is presented on Fig. 1.

Proper management in the pre-analytical phase is extremely important, as the toxin present in a stool sample is easily degraded at room temperature, and after about 2 h, it can be no longer detected in the acquired material. Once the stool sample is obtained, it should be stored at refrigerator temperature (+ 4 °C) and used for testing within the next 24 h [59]. The test is only performed on diarrheal sample unless ileus is suspected—in such case, it is acceptable to obtain a sample by rectal swab. With the exception of epidemiological purposes, it is not recommended to test stool samples obtained from asymptomatic patients. It is also not recommended to repeat testing for *C. difficile* after successful treatment is completed as there is a significant proportion of patients who test positive and their treatment does not need to be continued or repeated [18, 54, 60].

Endoscopic evaluation is also useful; however, it should be emphasized that it is not performed in patients with uncomplicated CDI that was confirmed with immunological tests. Endoscopy is indicated if diagnostic problems occur, namely, a typical CDI presentation with negative *C. difficile* test results, no response to standard course of antibiotics or when an alternative diagnosis is suspected, and direct visualization and/or biopsy of the bowel mucosa is needed. If the colonoscopy is performed, limited flexible sigmoidoscopy is preferred with minimal or no air insufflation to avoid perforation of the inflamed colon. The pseudomembranes found during the procedure are elevated, white to yellow lesions, typically about 2 cm in diameter, which are irregularly distributed and separated by normal mucosa. They are not removed by intestinal wall rinsing. The distribution of pseudomembranes tends to vary. Not all patients with CDI have pseudomembranes, and their absence does not rule out *C. difficile* infection. For example, pseudomembranes are rarely in recurrent CDI or in CDI among patients with inflammatory bowel disease [61–63]. On the other hand, pseudomembranous colitis can be caused by a number of different etiologies, like Behcet’s disease, collagenous colitis, inflammatory bowel disease, ischemic colitis, and also other infections, like CMV or enterohemorrhagic *Escherichia coli* O157:H7 [64].

Abdominal imaging (X-ray, ultrasound) in patients with CDI reveals distended bowel loops, often with wall thickening. Their use is of the highest importance when diagnosing CDI complications. Ultrasound imaging is an especially good method of monitoring the width of colon [18, 43]. Computed tomography of the abdomen and pelvis with oral and intravenous contrast is useful among patients with severe CDI, helping to evaluate for presence of toxic megacolon, bowel perforation, or other findings warranting surgical intervention [65].

Laboratory findings reveal high leukocytosis, elevated C-reactive protein, and in the most severe cases hypoalbuminemia as well as acute kidney injury [43].

## CDI treatment

### Vancomycin and fidaxomicin are the cornerstone of CDI treatment

Treatment should only be started in patients with CDI symptoms; presence of the *C. difficile* toxin without symptoms of the infection is not an indication for treatment. In 2014, the ESCMID guidelines were published in which two drugs metronidazole and vancomycin were the cornerstone of CDI treatment. Metronidazole was first-line drug in non-severe CDI, while vancomycin was the drug of choice for severe CDI [66]. Since then, the results of two identical, phase 3, multicenter, randomized, double dummy, double-blind, active-controlled, parallel-design efficacy studies (RCT) showed superiority of vancomycin relative to metronidazole. Clinical success occurred in 210 (81%) of 259 patients treated with vancomycin versus 202 (73%) of 278 patients who were treated with metronidazole (*p* = 0.02). However, among patients with severe disease, a statistically significant relationship was not achieved (clinical success achieved 78.5% in the vancomycin group compared with 66.3% in the metronidazole group (*p* = 0.059) [67]. In 2017, meta-analysis by Nelson et al. also concluded that metronidazole is inferior compared to vancomycin in the treatment of CDI [68].

Fidaxomicin is a drug that has been available since 2011. It is a macrocyclic, bactericidal antibiotic of narrow spectrum, directed primarily against Gram-positive pathogens. It has high efficacy against *C. difficile*, with no significant influence on the physiological flora of the colon. Fidaxomicin has efficacy comparable to vancomycin and in some groups higher effectiveness in reducing CDI recurrence. CDI recurrence after treatment of the first episode with fidaxomicin occurred in 15% of patients compared to 25% of patients treated with vancomycin. However, the same reduction in recurrence of the BI/NAP/027 strain was not observed [69, 70]. Fidaxomicin is also associated with lower percentage of CDI recurrence than vancomycin (20% vs 36%) in patients, who experienced CDI recurrence > 4 weeks after the treatment of the previous episode [71]. Another published meta-analysis suggested that fidaxomicin may be considered as first-line therapy for CDI [72]. Moreover, Guery et al. in 2017 showed that a tapered fidaxomicin treatment (days 1–5, 200 mg two times a day, followed by once daily on alternating days during days 7–25) is superior (*p* = 0.03) than vancomycin (125 mg oral capsules, four times daily on days 1–10) in resulting in a sustained clinical cure (30 days after end of treatment) in CDI. Recurrence rate at 90 day was also lower among fidaxomicin arm then vancomycin arm (9% vs 18%, respectively, *p* = 0,048) [73].

In 2017, Infectious Diseases Society of America (IDSA) and Society for Healthcare Epidemiology of America (SHEA) updated their guidelines, pointing that vancomycin and fidaxomicin are the cornerstone of CDI treatment [47] (Table 1).

**Table 1:** Antibiotic regimens used in the treatment of *C. difficile* infection [29, 47, 66, 67, 69–73]

First episode of the infection	
Non-severe disease	• Vancomycin 125 mg orally four times a day for 10 days
	OR
	• Fidaxomicin 200 mg orally twice a day for 10 days
	• If above agents are unavailable: metronidazole 500 mg orally three times a day for 10 days
Severe disease	• Vancomycin 125 mg orally four times a day for 10 days
	OR
	• Fidaxomicin 200 mg orally twice a day for 10 days
Fulminant disease (previously referred as severe complicated)	• Vancomycin 500 mg orally or via nasogastric tube four times a day
	AND
	• Metronidazole 500 mg IV 3 times a day + alternatively
	If ileus is present: vancomycin per rectum (vancomycin 500 mg in 100 ml saline as enema) four times a day* (10–14 days)
First recurrence	
If the first episode was treated with metronidazole or fidaxomicin:	
• Vancomycin 125 mg orally four times a day for 10 days	
If the first episode was treated with vancomycin:	
• Vancomycin pulsed-tapered orally (each dose 125 mg):	
# Four times daily for 10–14 days and then	
# Twice a day for 7 days, than	
# Once a day for 7 days, than	
# Every 2 or 3 days for 2–8 weeks	
OR	
• Fidaxomicin 200 mg orally twice a day for 10 days	
Second of subsequent recurrences	
• Vancomycin pulsed-tapered orally (regimen as above)	
OR	
• Fidaxomicin 200 mg orally twice a day for 10 days;	
OR	
• Vancomycin 125 mg orally four times a day for 10 days, followed by rifaximin 400 mg three times daily for 20 days	
OR	
• Fecal microbiota transplantation	

*If there is partial ileus, vancomycin should be administered both orally and rectally; if the ileus is complete, only rectal vancomycin should be used. Rectal vancomycin administration is associated with a risk of large bowel perforation, and should only be used in those patients who do not respond to oral therapy; some patients may have delayed response to treatment and clinicians should consider extending treatment duration from 10 to 14 days in such situations

There are no uniform criteria stratifying non-severe and severe CDI. Based on the commonly known risk factors for CDI severity and CDI criteria used by other researchers, we have proposed to define the severity of CDI as follows.

Severe CDI presents with or develops two or more of the following severity markers during the course of the disease: hypoalbuminemia (serum albumin < 3 g/dl), white blood cell count ≥ 15,000 cells/mm^3^, creatinine > 1.5 × baseline (or glomerular infiltration rate reduced by 25% from baseline), or temperature > 38.5 °C. Fulminant (severe complicated CDI), defined as CDI that presents with or develops at least one of the following signs or symptoms: admission to an intensive care unit, hypotension with or without use of vasopressors, ileus, toxic megacolon, mental status changes, serum lactate levels > 2.2 mmol/l, or any evidence of end organ failure [74].

If there is high suspicion of CDI with a negative ELISA assay, it is reasonable to start empiric antibiotic therapy for CDI. Other antibiotics that show activity against *C. difficile* include teicoplanin, tigecycline, bacitracin, and nitazoxanide. However, they are not included in CDI treatment recommendations and they may be considered when basic therapeutic options have run out. Pregnant and breast-feeding women should be treated with orally administered vancomycin in typical doses [3, 75–77]. The use of additional antibiotics (other than those treating CDI) is associated with increased risk of prolonged diarrhea and CDI recurrence, which is why they should be discontinued. However, if such therapy is indispensable, it should be continued preferably with antibiotics that are associated with lower risk of CDI, such as macrolides, aminoglycosides, sulfonamides, vancomycin, or tetracyclines. Some authors suggest that in this situation, prolonged treatment with antibiotics acting against *C. difficile* should be discontinued a week after the other broad-spectrum therapy is completed [78, 79]. According to authors of this paper, such regimen is worth implementing, if the additional CDI treatment does not exceed 7 to 10 days.

The role of probiotics in the treatment and prevention of CDI is completely unknown. Three large trials, including a meta-analysis have shown a positive effect of probiotics in the prevention of primary CDI. However, it should be emphasized that the meta-analysis has a variety of limitations. The studies included in the meta-analysis differ in regard to doses and types of probiotics, *C. difficile* strains, doses and types of antibiotics, time of therapy, and ultimately in some of these studies the number of CDI was very low in all study arms. Considering the pathophysiology of CDI, it appears that probiotics may be a part of CDI prevention or treatment; however, we still lack properly randomized studies addressing this problem [50, 80–84]. There are insufficient data at this time to recommend administration of probiotics for primary, secondary prevention or treatment of CDI [47, 66].

Asymptomatic *C. difficile* carriers have high concentrations of antibodies directed against toxins A and B [85]. Based on this knowledge, there are studies on intravenous administration of immunoglobulins as well as monoclonal antibodies that may be useful in both the treatment and prevention of CDI recurrences. Successful immune therapy for the treatment or prevention of CDI would be an interesting way of fighting the disease, as in contrast to vancomycin, metronidazole, and fidaxomicin, it would not disrupt the bacterial flora of the host [86]. Anti-toxin antibodies are of great importance in the development of immunity against CDI. It has been shown that naturally produced anti-toxin antibodies in *C. difficile* colonized patients who did not develop diarrhea have protective effects [87, 88]. The administration of monoclonal antibodies directed against these toxins markedly reduced the risk of CDI recurrence. Thus, there is a chance that in the near future, we will have new forms of protection against *C. difficile* recurrence which might even be used when fighting the first infection [89]. Bezlotoxumab (a monoclonal antibody that binds to *C. difficile* toxin B) was approved by the FDA in 2016 for prevention of recurrent CDI in patients with high risk of CDI recurrence. The registration trial, which included over 2500 patients, showed that bezlotoxumab together with standard oral antibiotic therapy was associated with a significantly lower rate of recurrent infection than oral antibiotic therapy alone (17 versus 28%). At the same time, no similar effect was shown for actoxumab (a monoclonal antibody that binds to *C. difficile* toxin A). Bezlotoxumab is undoubtedly a significant achievement in CDI prevention; however, its use is limited by high cost and potential side effects. In the group of patients treated with bezlotoxumab, the incidence of acute decompensated heart failure was significantly higher when compared with the placebo group (12.7% vs 4.8% respectively) [90].

## Fecal microbiota transplantation

### Fecal microbiota transplantation have the highest rate of prevention of recurrent CDI among all therapeutic options

The fecal microbiota transplantation (FMT) procedure has been known for over 1000 years, and was first described by a traditional Chinese medicine doctor Ge Hong, who lived during the Dong Lin Dynasty period (284–364 BC). He applied human fecal suspension orally to patients with severe diarrhea or food poisoning [91]. In Europe, the idea was first used in veterinary medicine by the Italian anatomist Fabricius Aquapendente in the seventeenth century [92]. Modern medicine first performed fecal transplantation in 1958. Eiseman et al. used fecal enema as therapy for pseudomembranous enterocolitis (*C. difficile* had not been routinely identified at that time) [93]. The first report about fecal transplantation in patient with confirmed *C. difficile* infection was published in 1983 [94].

Nowadays, abnormal gut microbiota is considered a key factor in CDI development. Relatively short antimicrobial therapy might dramatically reduce the amount of intestinal microbiota, but recovery may last several months. During that specific period, patients lack their protective barrier, and with exposure to spores an infection might develop quickly. Even though the gut microbiota is composed of thousands of species of microbes, it is thought that *Bacteroides* and *Firmicutes* play predominant role in immunological responses against *C. difficile* [95].

Antibiotic withdrawal together with FMT have the highest rate of prevention of recurrent CDI among all therapeutic options [96–98]. Nood et al. performed open-label, randomized, controlled trial, which compared three treatment regimens: the infusion of donor feces preceded by an abbreviated regimen of vancomycin and bowel lavage, a standard vancomycin regimen, and a standard vancomycin regimen with bowel lavage. The study was stopped after an interim analysis. Eighty-one percent of the patients in FMT group had resolution of CDI comparing with only 31% patients receiving vancomycin alone and 23% receiving vancomycin with bowel lavage (*p* < 0.001 comparing with both control group). Moreover, no significant differences in adverse events among the three study groups were observed except for mild diarrhea and abdominal cramping in the FMT group on the infusion day. After FMT, patients showed increased fecal bacterial diversity, similar to that in healthy donors [98]. One hundred percent effectiveness was observed in the 27-patient study by Dutta et al. where fecal material was directly introduced into small and large intestine [96]. Louie et al. performed interesting experiments with fecal microbes contained in gelatin capsules. According to the protocol, 27 patients received 24–34 capsules, and the effectiveness of the therapy was also 100% [97].

Transplantation with frozen fecal material, first described by Borody and Khoruts, simplified the procedure [99]. Stool samples can be stored at − 80 °C and used during next 5–6 months. Some stool banks extend storing period for 2 years [100]. FMT procedure has not yet been standardized. The donated stool is mixed with normal saline solution, homogenized, and filtrated to separate the solid parts, to obtain fluid material. Fecal transplant can be administrated via oral capsules, lower gastrointestinal (GI) tract procedure (colonoscopy, retention enema), or upper GI tract procedure (nasojejunal/nasoduodenal tube) [101]. Some potential complications of FMT are connected with delivery method (e.g., perforation with colonoscopy, aspiration pneumonia with upper GI administration). However, the frequency of complications associated with FMT is likely similar to the frequency of complications when these procedures are performed for other indications. It seems that the effectiveness of FMT is higher after lower GI tract administration when compared to upper GI tract administration [101]. Retention enema is a procedure with a low cost, easily accessible, and with relatively low risk of complications; however, it may be difficult to maintain stool transplant and in such a situation, patients require repeated enema [101]. FMT is highly promising treatment of CDI, and several large “feces banks” have been developed. In the Netherlands, treatment with FMT is organized at a national level by the “Netherlands Donor Feces Bank” (NDFB, https://www.ndfb.nl/) at Leiden University Medical Center. Since 2016, NDFB has received more than 120 requests for treatment of FMT for patients with recurrent CDI. Each request is discussed by a panel of experts comprising of medical microbiologists, gastroenterologists, and infectious disease physicians. Of all received requests, only 80% fulfilled to the criterium of recurrent CDI and were treated. The success rate was very high, more than 90%, and clearly associated with the stringent inclusion criteria (E.T Terveer,. K.E Vendrik and E.J.Kuijper, manuscript in preparation). Two main concerns are the risk of transferring infectious pathogens from the donor to the recipient, and development of autoimmunological disorders. Although the possibility of infecting the recipient with fecal material still exists, it is minimalized by proper donor screening. Potential donors should be healthy, have daily formed bowel movement, and screened for bacterial, viral, and parasites infection, as it is presented in thorough Terveer et al. study [100]. The influence of gut microbiota on some immune-mediated diseases such as irritable bowel disease raises concerns about long-term side effects of fecal transplantation. This field merits further investigation in the future [86].

## Conclusion

In the past decade, CDI became one of the most detrimental nosocomial infections. It is of most importance to remember that CDI prevention starts with healthcare professional education regarding such preventive measures, as hand washing, gloves wearing, proper decontamination of medical devices and patient’s environment, as well as optimal antibiotic management. Hospitalized elderly patients treated with antibiotics are at the highest risk for CDI. FMT is highly promising treatment of CDI.

## Abbreviations

CDI: *Clostridium difficile* infection
CDT: binary toxin
CYTA: cytotoxic assay test
*C. difficile*: *Clostridium difficile*
EIA: enzyme immunoassay
FMT: fecal microbiota transplantation
GDH: glutamate dehydrogenase
GI: gastrointestinal
ICU: intensive care unit
NAAT: nucleic acid amplification test
